# Well Done!

**Published:** 1886-10-09

**Authors:** 


The Hospital.

**An Institution, Family, and Parochial Journal of Hospitals, Asylums, and all Agencies for the Care of the Sick, Criticism and News.**

### WELL DONE!

**The First Time.** THE Lord Mayor, to whose popularity, personal labours, and devotedness the result is largely due, has had the satisfaction to announce that the collections made in the churches and chapels throughout the Metropolis on Hospital Sunday in June last amounted to £40,000. This amount does not include the late Dr. Wakley's contribution of £1,000, and there are still some fifty-four churches and chapels which have yet to pay in the sums collected on Hospital Sunday. This is the first time that anything approaching £40,000 has been raised in connection with this fund. The largest sum contributed by the congregations in any former year was £34,830, in 1884, to which was afterwards added £4,500, as the proceeds of the Hospital *Fête* at the International Health Exhibition. This result has been attained without any increase in the cost of collection, which will probably amount to less than 3½ per cent. of the gross receipts. Of course, such a result has not been attained without considerably increased labour on the part of the officers of the fund. The thanks of everyone interested in the hospitals of London are especially due to the Secretary, Mr. Henry N. Custance, for the cheerful way in which he has undertaken much extra labour at a time when the medical charities of the Metropolis were suffering greatly from want of adequate pecuniary support.

**How the Result was Obtained.** This year, by the institution of "The Hospitals Week," an opportunity was afforded to all classes, from the highest to the lowest, and to all professions, to politicians and journalists, as well as to physicians and the clergy, to lend a hand with the object of awakening London to its responsibilities and duties with regard to this question. The Metropolitan daily press, notwithstanding the political crisis and the great pressure on their columns, devoted each day at least half a column, and sometimes more than a column, to reports of the meetings held in all parts of London. The Presidents at the meetings were Sir Andrew Clark, Bart., M.D.; Lord Dartmouth; the Duke of Norfolk; Lord Northbrook; the Duke of Westminster; and Mr. E. R. Cooke, M.P. The speakers included H.R.H. the Duke of Cambridge; the Marquis of Salisbury; Lord Cadogan; the Right Hon. W. H. Smith, M.P.; Baron F. de Rothschild, M.P.; Baron Henry De Worms, M.P.; Lord Charles Beresford, M.P.; Sir

Julian Goldsmid, M.P.; Sir Edmund Currie; Sir Risdon Bennett, F.R.S.; Sir Sydney Waterlow, Bart., M.P.; Sir William Mac Cormac; The Bishop of Colchester; Cardinal Manning; the Rev. Newman Hall; the Rev. G. S. Reaney; many Members of Parliament, and other gentlemen of influence too numerous to mention. A debt of gratitude is due to all these gentlemen who worked with a will on behalf of the hospitals. To the proprietors of the *Lancet*, and especially to Dr. Wakley's brother, Mr. Thomas Wakley, upon whose shoulders much anxiety and trouble were cast by the publication of the three Special Supplements of the *Lancet*, which did so much to arouse London, very special thanks are due. No one could have worked harder than Mr. Wakley and his assistants to secure a successful result, and without this aid we feel the £40,000 could never have been raised. Besides, the three Special Supplements must have cost hundreds of pounds, and their publication and free distribution by the proprietors of the *Lancet* was an act of public spirit, that ought to be widely recognised and known.

**The Special Difficulties of the Year.** The Council of the Hospital Sunday Fund determined to take this new departure long before it was possible to foresee that a general election would be precipitated in the middle of June. The nature of the financial depression, and its effect upon all classes, had convinced the Council that there was sufficient reason to warrant them in a supreme effort to awaken the many to the privilege and duty of giving something on Hospital Sunday. When all the arrangements were far advanced, it became certain that a dissolution of Parliament would take place in the very week which had been set apart as The Hospitals Week. One result of this was the absence of many influential and rich people from the Metropolitan churches on the day of the collections. Owing, however, to the self-sacrifice and loyal determination of the eminent men whose names we have recorded as being present at the various meetings, representative speakers and speakers of eminence were in attendance each day, and each day the interest of the general public, despite the political excitement, steadily grew and increased. It was felt more and more, as the facts became known, that the laity owed it to themselves to co-operate with the clergy in making the Metropolitan Hospital Sunday Fund collection worthy of London. The result has shown that, when the inhabitants of the greatest city of the Empire are appealed to in the right way, they are neither more nor less wanting in public spirit and generosity than the inhabitants of other cities and towns throughout the English-speaking world.

**The Action of the Hospitals.** There was at one time an unexpected difficulty, caused by the narrow view taken by a few hospital committees of this new departure on the part of the Hospital Sunday Fund Council. It is not surprising, although it is very gratifying to be able to record, that this opposition to the Hospitals Week was so limited in character as to be really beneficial, by exciting the enthusiasm of those far-seeing hospital managers who took an opposite view. It is a pleasant duty to place on record the names of the hospital officials, and of the institutions which they represent, who laboured hard to raise a larger sum on Hospital Sunday, 1886, than had ever been raised before. The Council of the Hospital Sunday Fund have greatly appreciated the large amount of time which each of the following gentlemen, and many others connected with the institutions mentioned, so cheerfully gave up to the work. We understand each of them has received a *souvenir* from the Mansion House, which we make no doubt they will greatly value:—Mr. W. T. Grant, Great Northern Hospital; Mr. H. Haggard, London Hospital; Mr. W. J. Purver, Miller Memorial Hospital; Mr. Pietro Michelli, St. Mary's Hospital, Paddington; Mr. W. T. Evans, Seamen's Hospital; Mr. A. O'D. Bartholeyns, Middlesex Hospital; Captain Storar Smith, City of London Hospital for Diseases of the Chest; Mr. J. J. Austin, Royal Hospital for Diseases of the Chest, City Road; Mr. R. A. Owthwaite, City of London Lying-in Hospital; Mr. T. Ryan, Queen Charlotte's Lying-in Hospital; Mr. R. G. Kestin, Royal Hospital for Children and Women; Mr. E. Hopwood, M.D., London Fever Hospital; Mr. H. Canning, National Orthopædic Hospital; the Secretary, Camberwell Dispensary; the Secretary, Battersea Dispensary; Mr. C. Bartram, Islington Dispensary; and Mr. St. Leger Bunnett, St. George's and St. James's Dispensary.

**The Future.** The events above recorded are satisfactory in every way, and reflect great credit upon all concerned. We feel, however, that a sum approaching £80,000 ought to be raised in the Metropolis on Hospital Sunday. The results which have attended the establishment of the Hospitals Week strengthen the belief that if a similar organisation be continued from year to year, such a sum as we have named will one day most certainly be raised in the churches and chapels of London.

